# Tympanal ears mediate male–male competition, courtship and mating success in *Bicyclus anynana* butterflies

**DOI:** 10.1098/rsos.231386

**Published:** 2024-03-27

**Authors:** Galen J. L. Tiong, Lin Naing, Edwin Ng, Emilie Dion, Antónia Monteiro

**Affiliations:** ^1^ Department of Biological Sciences, National University of Singapore, 14 Science Drive 4, Singapore 117543, Republic of Singapore

**Keywords:** *Bicyclus anynana*, hearing, Vogel’s organ, courtship, competition, seasonal plasticity

## Abstract

The presence of intra-specific acoustic communication in diurnal butterflies is not well established. Here, we examined the function of the tympanal ear (Vogel’s organ, VO) in the seasonally polyphenic butterfly *Bicyclus anynana* in the context of sexual signalling. We investigated how the VO and the flanking enlarged veins, which are suggested sound resonance chambers, scale with wing size across sexes and seasonal forms, and how disruptions to the VO alter courtship behaviour and mating outcomes. We found that males have VOs similar in size to females despite having smaller wings, and dry season (DS) male cubital and anal veins do not scale with the wing size. This suggests that the VO plays an important role in males and that cubital and anal veins in DS males may be tuned to amplify specific sound frequencies. Behavioural assays performed with deafened and hearing males of different seasonal forms, in pair and triad settings, showed that deafened DS males, but not wet season males, experienced lower mating success relative to their hearing counterparts. Our study documents a novel function for the wing tympanal membrane in mediating courtship and mating outcomes in diurnal butterflies.

## 1. Introduction

Acoustic communication in insects is a core component of species recognition, sexual attraction and courtship in many species. Certain insect taxa, such as orthopterans, cicadas and some groups of moths, produce and use far-field sound to attract and locate a suitable mate over long distances [1–3]. Near-field sound, on the other hand, decays rapidly and is mostly used for intimate communication between conspecifics [4,5]. Arthropods that produce near-field courtship songs for mate attraction include whip spiders, which vibrate their antenniform legs [6], wolf spiders, which produce rapid leg waving [7], and *Drosophila*, which vibrate their wings to court females [4]. Courtship songs and other acoustic signals in insects can also induce specific courtship behaviours in conspecifics of the same sex, often the males. *Drosophila* males, for example, can respond to courtship song by courting other conspecific males while extending their wings, often resulting in a chain of courting males called the male chaining response [8]. Black soldier fly *Hermetia illucens* males are stimulated by acoustic signals produced during flight by both male and female conspecifics to engage in wing fanning and mounting [9]. The presence of a same-sex rival can also modulate the structure of courtship song produced by a courting insect, like in courting *Drosophila* males [10]. These studies show that both far- and near-field sounds, and the presence of conspecifics, can impact species-specific courtship behaviours.

In Lepidoptera, the study of acoustic communication in courtship has primarily been focused on nocturnal moths of the Pyraloidea and Noctuoidea families [2]. Male moths broadcast ultrasonic courtship songs produced by their tymbal abdominal organs or via the stridulation of specialized wing scales [11–14]. These signals are perceived by female tympanal hearing organs tuned to such frequencies [2,5,15]. Male song production in these species is vital for females to recognize males and accept mating. Muting sound-producing organs [12,14,16], or perforating tympanal membranes in females [11,17,18], drastically reduces the moths’ mating success, demonstrating the critical role of acoustic signalling in mate choice. Males can also use distinct ultrasonic signals to communicate with conspecific males when engaging in agonistic rival behaviour [12,15,19], which can serve to disrupt rival courtship and mating attempts [20]. These experiments show that these moths are sensitive to ultrasonic sounds and that both sexes can perceive these sounds and alter their sexual behaviour.

The role of tympanal ears and acoustic communication in butterflies is less well understood. A few studies, however, have examined the morphology and neurophysiology of tympanal ears and associated response behaviours in a selected few crepuscular [21,22] and diurnal [23–29] butterfly species. The tympanal hearing organ in butterflies, also called the Vogel’s organ (VO) [30], is a unique structure characterized by a thin cuticular membrane and its associated chordotonal organ located at the base of the forewing cubital vein [21,24,27,31]. In species with a VO, the proximal part of this cubital vein, as well as that of two other flanking wing veins, the subcostal and anal, are often enlarged. These inflated wing veins, found primarily in the tribe Satyrini and in the subfamily Biblidinae [32], provide butterflies a mechanism for auditory low-frequency tuning. They can be used as resonators to amplify specific frequencies of sound, as dampeners to reduce ambient noise or as conduits to transmit sound to the ear [25,32,33]. In *Hamadryas chloe* and *Yphthimoides castrensis*, the subcostal vein functions as a resonance chamber for clicking sounds produced by vein calluses striking against each other during wing fluttering [34,35]. These sounds were proposed to function in male agonistic interactions or courtship behaviour [29,31,35–38]. Both butterfly species also possess well-developed VOs, which were hypothesized to detect conspecific clicks in *Hamadryas feronia*. However, experimental manipulations of these organs to determine their biological function have never been attempted [31]. In the common wood nymph *Cercyonis pergala*, inflated veins increase the sensitivity of the VO to low-frequency sounds (via acoustic impedance matching) [25], leading to the hypothesis that the VO might be used to detect low-frequency sounds such as avian predator flight sounds [24,27] and/or predator calls [23,26]. As most species of diurnal butterflies are not known to produce sound for communication or to have tympanal ears tuned to detect conspecifics, sound production has largely been overlooked in favour of stereotypic visual and pheromonal signalling [39–41]. In addition, the role of VO in intra-specific communication remains to be investigated in butterflies.

**Figure 1.**
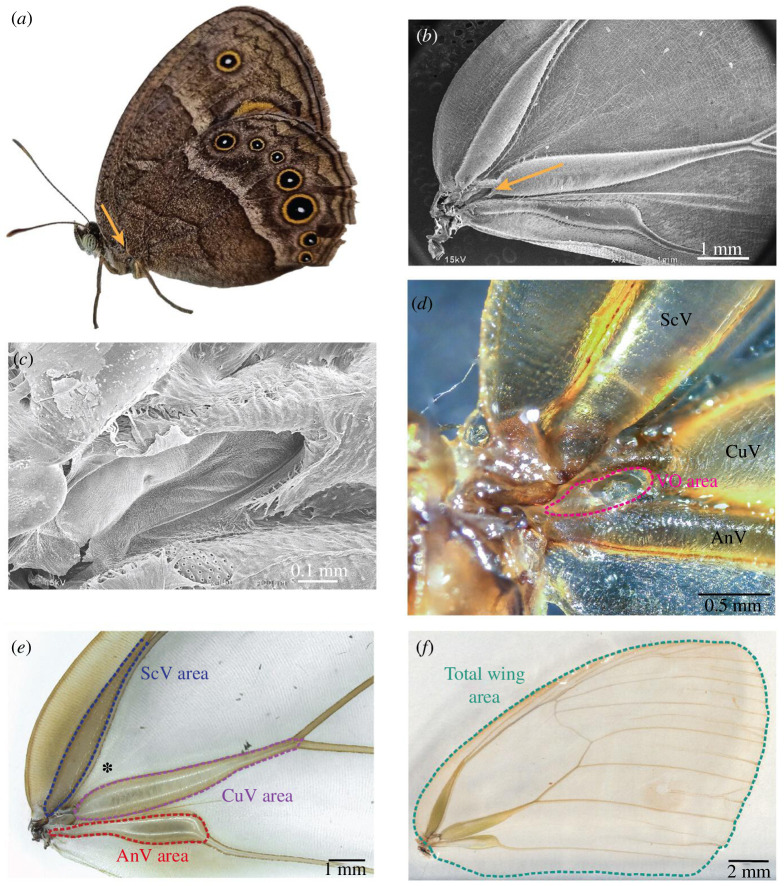
Vogel’s organ ( VO ) and wing vein measurements and manipulations performed in *Bicyclus anynana* butterflies. (*a*) The VO is located on the forewing at the base of the cubital vein in both males and females (orange arrow). (*b,c*) Scanning electron microscopy images of the VO. The VO, veins and total wing areas were measured following the dashed line patterns shown in (*d*–*f*), respectively. The asterisk in (*e*) indicates the location of the sham male wing perforation. All images are from females, and all (except *a*) are from descaled wings. ScV, CuV and AnV are the the subcostal, cubital and anal veins, respectively.

Here, we explore the role of sound in intra-specific signalling in the model butterfly *Bicyclus anynana* (Nymphalidae: Satyrinae). This species uses visual and olfactory sexual signals for intra-specific signalling, but sound signals have never been explored. *B. anynana* is a seasonal polyphenic species that demonstrates plasticity in courtship behaviour, in levels of male sex pheromone production and in the size and brightness of the UV-reflective centres of eyespot colour patterns, which function as sexual ornaments [42–44]. Specifically, these butterflies show a reversal of sex roles between seasonal forms. Wet season (WS) males actively court WS females, whereas dry season (DS) males court less and are in some cases courted by DS females [43]. Courtship routines involve the courter orienting its body towards the opposite sex, in close proximity and rapidly opening and closing its wings [45]. Wing flickering exposes the dorsal wing eyespots [43,46] and also helps release sex pheromones from glandular wing organs in males [39,44,45]. Such flickering might also produce an acoustic signal, but sensitivity to sound in this species has never been investigated. However, an initial examination of *B. anynana* wings revealed an organ with a membranous component at the base of the cubital forewing vein (figure 1) that is anatomically like the VO characterized in other butterflies [21,27,28,32], suggesting that the species might be using it to perceive sounds.

We began this study by examining the morphology of the VO and enlarged wing veins in both males and females of both seasonal forms. Guided by our measurements (see Discussion), we subsequently hypothesized that the VO may play a key role specifically in male *B. anynana* and decided to examine how the VO may be mediating both male–female and male–male communication in the context of male competition for mating opportunities. We tested this hypothesis by disrupting the tympanal membrane in males and measuring male behaviour in both single-pair interactions and in male–male competitive situations. Given the seasonal plasticity in *B. anynana* male courtship behaviour [43], we also hypothesized that the effect of VO impairment would have distinct effects on male courtship behaviour in *B. anynana* WS and DS seasonal forms.

## 2. Methods

### 2.1. Animal husbandry

We reared *B. anynana* butterflies in climate-controlled rooms with 60% humidity, 12:12 h light:dark photoperiod, at 17°C and 27°C to induce DS and WS phenotypes, respectively. *B. anynana* larvae were fed young corn plants, and adults were fed mashed bananas. Animals used for measurements were frozen upon emergence. Animals used for behavioural experiments were sexed as pupae and separated by sex into different cages. Upon eclosion, butterflies were transferred into cages containing cohorts of the same eclosion day. We used 3- to 6-day-old naive virgin male and female butterflies in all the behavioural experiments.

### 2.2. Scanning electron microscopy

Wings were descaled by dipping in 95% ethanol, then in 100% bleach solution, followed by 95% ethanol, for 2 min each dip, dried, then mounted on aluminium stubs and sputter-coated with a thin layer of gold using a JEOL JFC 1100 ion sputter. The samples were imaged with a JEOL JSM 6510 scanning electron microscope.

### 2.3. Measurements of Vogel’s organ and wing vein areas

We measured the area of the VO and the enlarged area of the wing veins adjacent to the VO in *B. anynana* males and females of both seasonal forms. Right forewings from 52 DS (32 females and 20 males) and 53 WS (24 females and 29 males) butterflies were excised with fine scissors and descaled as described above. Descaled wings were floated in a Petri dish filled with 95% ethanol and images were captured with a Leica DMS1000 microscope camera. VO area, total wing area and all three wing vein areas (subcostal, cubital and anal) were measured using the magnetic lasso tool in Adobe Photoshop (figure 1).

### 2.4. Allometric analyses

To visualize how the VO area scaled with the wing size, we plotted the log of the VO area (in mm^2^) as a function of the log of wing area (in mm^2^) and then tested whether the intercept and slope of the regression line fitted to the data varied with sex and seasonal form using linear models. Vein measurements were analysed similarly; additional analyses were performed for males and females separately to compare seasonal differences in wing vein allometry between sexes without sacrificing statistical power by considering three-way interactions. For each vein, we constructed linear models of log vein area (in mm^2^) as a function of log wing area, sex and seasonal form. We also constructed linear models separately for each sex. All possible three-way and two-way interaction factors were tested using ANOVA and were only retained in the model if significant. Estimated marginal means (average size of a trait for an average sized wing) and contrasts were generated from fitted models (electronic supplementary material, table S1). Model assumptions were verified by plotting residuals versus fitted values for each covariate in all models. All statistical analyses were performed in R 4.2.1 [47] and RStudio [48]; the ‘emmeans’ package [49] was used for generating estimated marginal means and contrasts.

### 2.5. Hearing impairment behavioural experiments

Behavioural experiments were conducted in a 24°C, 60% humidity environment in cylindrical hanging net cages (30 cm diameter × 40 cm height) under full spectrum (Plantmax) and UV (Arcadia Marine Blue) lights. To prevent mate choice copying [50], cages were visually isolated from one another by pieces of green opaque cloth. All observations were done manually between 15.00 and 18.00 over several weeks, with two to four cages being observed at a time. To determine the role of the male tympanal ears in mediating courtship intensity and mating success, we tested whether impairing hearing in *B. anynana* males impacted male behaviour, both in the absence and in the presence of a male competitor. Treatment males (henceforth referred to as ‘deafened’) were prepared by securing forewings with flat forceps and perforating the tympanal membrane under a stereoscopic microscope with a glass microcapillary needle (prepared with Flaming/Brown micropipette puller, Model P-97). Sham males (referred to as ‘hearing’) were prepared by perforating basal forewing tissue ~1 mm adjacent to the ear without damaging either the tympanal membrane or wing veins (see figure 1*e*).

#### 2.5.1. Pair settings

We first investigated the impact of tympanal ear impairment on male courtship behaviour in a pairwise context (one male and one female), where males were paired with females of the same seasonal form. We set up a 2 × 2 factorial design with male treatment (deafened or hearing) and butterfly seasonal form (WS or DS) as variables. We tested 15 butterfly pairs with each treatment/season combination for a total of 60 trials. Males were operated on and released into their respective experimental cages and allowed to acclimate for 30 min before the start of the experiment. We then released one female into each cage to start the experiment. We subsequently recorded all courtship behaviours until successful copulation or until 2 h had elapsed. We recorded the duration of individual courtship bouts, defined as the start of wing flickering/attempted copulation to interruption by either party (i.e. flying or walking away) or successful copulation, the number of courtship bouts, total courtship duration (the sum duration of all courtship bouts within a trial) and latency to mate (from the start of the experiment to successful copulation).

#### 2.5.2. Triad settings

We evaluated the impact of hearing on butterfly behaviour in a male–male competition setting by conducting triad experiments. Triads consisted of one deafened male and one hearing male of the same age, as well as one female. Both males were operated on and released into the same cage to acclimate for 30 min before the start of the experiment when a single female was introduced. We alternated introducing deafened or hearing males into the experimental cage first between each trial. To distinguish deafened and hearing males, we marked both hindwings with either one or two dots with a permanent black Sharpie marker. The type of marking was alternated for deafened and hearing males between trials. We recorded the same courtship metrics as for the pair experiments, as well as mating outcome, that is, the identity of the first male to mate successfully. The behavioural assay lasted until the first mating occurred or for a maximum of 2 h.

### 2.6. Statistical analysis of behavioural data

We analysed behavioural data from pair and triad experiments separately, focusing on differences in durations of individual courtship bouts, number of courtship bouts, total courtship duration and latency to mate. For triad scenarios, the number of courtship bouts and total courtship duration were determined for each male. We compared these data using a Hurdle model [51], first comparing the proportions of males of each treatment and seasonal form that engaged in courtship, and subsequently comparing only non-zero durations and counts between males. Latency to mate in triad trials was determined only for the males that mated successfully. We constructed regression models for courtship bout duration (in seconds), number of courtship bouts, total courtship duration (in seconds), latency to mate (in seconds) and likelihood to court (triads only), each as a function of treatment, seasonal form and their interaction, if significant. Differences in likelihood to court and mating success between hearing and deafened males in triads were determined using chi-squared and exact binomial tests for WS and DS seasonal forms. All statistical analyses were performed in R 4.2.1 [47] and RStudio [48] with the ‘MASS’ package [52] used to construct negative-binomial generalized linear models (table 2b,f), and the ‘multcomp’ package [53] used to perform general linear hypothesis testing on regression models.

**Table 1.** Summary of regression models for courtship behaviour metrics from pair (a–d) and triad (e–h) experiments. Behavioural metrics evaluated were courtship event duration (a,e), number of courtships (b,f), total courtship durations (c,g), and latency to mate (d,h), with treatment (categorical with two levels: control males (hearing; 0) and hearing-impairment (deaf; 1)), seasonal form (categorical with two levels: DS ( d ry season ; 0) and WS ( w et season ; 1)) and their interaction, if significant, as variables. The normality of data was tested using Shapiro–Wilk tests. Courtship event duration and total courtship duration data were log-transformed, and latency to mate data were square-root-transformed to best approximate normal distributions before analysis with linear regression. A number of courtship bouts were analysed using a generalized linear model with a negative binomial distribution, which is appropriate for our overdispersed count data. Both original (‘*p*’) and multiplicity-corrected (using Benjamini–Hochberg (‘false discovery rate(FDR)’) method; ‘*p* (FDR)’) *p*-values are presented; tested *p*-values were corrected within pair (a–d; electronic supplementary material, table S3; *n* = 10) and triad (e–h; *n* = 9) model batches. *p*-values <0.05 are in bold.

predictors	estimates	95% CI	*p*	*p* (FDR)	estimates	95% CI	*p*	*p* (FDR)
	(a) courtship duration (log s), pair				(e) courtship duration (log s), triad			
(intercept)	2.56	2.28–2.83			1.70	1.47–1.94		
treatment (deaf)	−0.36	−0.75–0.03	0.067	0.152	0.06	−0.23–0.35	0.683	0.769
season (WS)	−0.44	−0.84 to −0.03	**0.034**	0.114	0.27	−0.02–0.56	0.063	0.285
treatment (deaf) × season (WS)	1.14	0.52–1.76	**<0.001**	**0.004**				
observations	229				301			
*R* ^2^	0.056				0.012			
*R* ^2^ adjusted	0.044				0.005			
	(b) number of courtships, pair				(f) number of courtships, triad			
(intercept)	1.65	1.28–2.03			1.19	0.93–1.45		
treatment (deaf)	−0.26	−0.70–0.18	0.251	0.279	0.16	−0.16–0.49	0.323	0.562
season (WS)	−0.31	−0.75–0.13	0.169	0.223	−0.08	−0.40–0.25	0.641	0.769
observations	57				88			
*R* ^2^ Nagelkerke	0.082				0.022			
	(c) sum courtship duration (log s), pair				(g) sum courtship duration (log s), triad			
(intercept)	3.89	3.43–4.36			3.24	2.89–3.59		
treatment (deaf)	−0.04	−0.58–0.50	0.881	0.881	−0.04	−0.50–0.42	0.868	0.868
season (WS)	0.40	−0.14–0.94	0.147	0.223	0.38	−0.08–0.84	0.103	0.310
observations	57				88			
*R* ^2^	0.039				0.031			
*R* ^2^ adjusted	0.003				0.008			
	(d) sqrt latency to mate (in seconds), pair				(h) sqrt latency to mate (in seconds), triad			
(intercept)	36.72	25.93–47.51			37.22	25.96–48.48		
treatment (deaf)	−8.53	−21.07–4.01	0.178	0.223	−6.76	−21.95–8.42	0.375	0.562
season (WS)	11.32	−1.22–23.85	0.076	0.152	8.45	−5.48–22.37	0.229	0.514
treatment (deaf) × season (WS)					24.33	5.00–43.66	**0.015**	0.132
observations	55				52			
*R* ^2^	0.089				0.366			
*R* ^2^ adjusted	0.054				0.326			

## 3. Results

### 3.1. Vogel’s organs have similar absolute sizes across *B. anynana* sexes and seasonal forms

**Figure 2.**
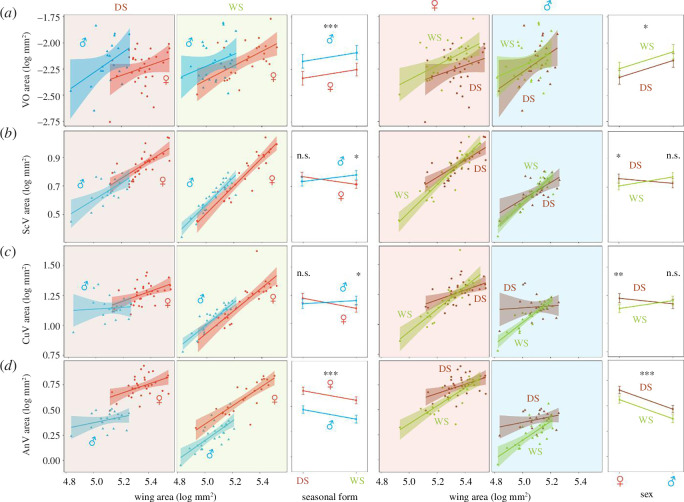
Male *B. anynana* have larger VO relative to females of the same size; cubital and anal wing vein sizes do not scale with the wing size in DS males. Left and right graph subsets compare morphological differences for log sizes of (*a*) VO, (*b*) subcostal (ScV), (*c*) cubital (CuV) and (*d*) anal veins (AnV) between male (*n* = 49) and female (*n* = 56) *B. anynana*, and between dry season (DS; *n* = 52) and wet season (WS; *n* = 53) *B. anynana*, respectively. Within subsets, left and centre graphs depict the allometric relationship between log area and log wing area faceted either by DS and WS phenotypes (for male/female comparison) or by males and females (for a DS/WS comparison); right graphs depict differences in estimated marginal means for the trait for a fixed log wing area. Best fit lines and confidence intervals are red for females (♀), blue for males (♂), green for WS and brown for DS; male and female *B. anynana* are represented with triangles and circles, respectively. Error bars represent 95% confidence intervals; *p*-values >0.05, <0.05, < 0.01 and < 0.001 are represented as ‘n.s.’, ‘*’, ‘**’ and ‘***’ respectively.

The absolute area of VOs did not differ between sexes (*t*‐test; *p* = 0.430) and seasonal forms (*t*‐test; *p* = 0.160). There were no significant two-way or three-way interactions between VO area, sex and seasonal form (figure 2*a*, table 2a). Female *B. anynana* have larger wings than males (Welch two-sample *t*‐test; *p* < 0.001), while DS butterflies have larger wings than their WS counterparts (*t*‐test; *p* = 0.004). Larger wings have larger VOs across all groups (table 2a; *p* = 0.042, *p* (FDR) = 0.051). VOs also have a negative allometry with respect to wing size (electronic supplementary material, table S1a; log wing area estimate = 0.64). This translates to males having significantly larger VOs than females for the same wing area (figure 2*a*, table 2a; *p* = 0.001, *p* (FDR) = 0.003); and WS butterflies having larger VOs compared with their DS counterparts for the same wing area (figure 2*a*, table 2a; *p* = 0.015, *p* (FDR) = 0.023).

**Table 2.** ANOVA tables of allometric linear regression models for log VO and wing vein (subcostal (ScV), cubital (CuV) and anal (AnV)) areas. VO and wing vein models (a) were run with log wing area, seasonal form, sex and their interactions, if significant, as variables. Additional wing vein models were run for female (b) and male (c) *B. anynana* separately, with log wing area, seasonal form and their interaction, if significant, as variables. Both original (‘*p*‐value’) and multiplicity-corrected (using Benjamini–Hochberg (‘FDR’) method; ‘*p*‐value (FDR)’) *p*-values are presented; tested *p*-values were corrected within the full model (*n* = 17), female-only model (*n* = 9) and male-only model (*n* = 9) batches. *p*-values <0.05 are in bold.

	df	Sum Sq	Mean Sq	*F* value	*p*‐value	*p*‐value (FDR)
**(a)**	**VO area (log mm^2^)**					
log wing area	1	0.121	0.121	4.232	**0.042**	0.051
sex	1	0.312	0.312	10.887	**0.001**	**0.003**
season	1	0.176	0.176	6.164	**0.015**	**0.023**
residuals	101	2.891	0.029			
	**subcostal vein area (log mm^2^)**					
log wing area	1	1.773	1.773	481.526	**<0.001**	**<0.001**
sex	1	0.008	0.008	2.268	0.135	0.153
season	1	0.001	0.001	0.275	0.601	0.601
log wing area × season	1	0.016	0.016	4.312	**0.040**	0.051
sex × season	1	0.027	0.027	7.283	**0.008**	**0.015**
residuals	99	0.364	0.004			
	**cubital vein area (log mm^2^)**					
log wing area	1	1.212	1.212	196.799	**<0.001**	**<0.001**
sex	1	0.008	0.008	1.346	0.249	0.264
season	1	0.026	0.026	4.294	**0.041**	0.051
log wing area × season	1	0.096	0.096	15.639	**<0.001**	**<0.001**
sex × season	1	0.042	0.042	6.782	**0.011**	**0.018**
residuals	99	0.610	0.006			
	**anal vein area (log mm^2^)**					
log wing area	1	4.081	4.081	498.388	**<0.001**	**<0.001**
sex	1	0.401	0.401	48.935	**<0.001**	**<0.001**
season	1	0.170	0.170	20.738	**<0.001**	**<0.001**
log wing area × season	1	0.080	0.080	9.805	**0.002**	**0.005**
residuals	100	0.819	0.008188			
**(b)**	**subcostal vein area (log mm^2^), female**					
log wing area	1	0.635	0.635	157.197	**<0.001**	**<0.001**
season	1	0.001	0.001	0.238	0.628	0.628
log wing area × season	1	0.027	0.027	6.630	**0.013**	**0.019**
residuals	52	0.210	0.004			
	**cubital vein area (log mm^2^), female**					
log wing area	1	0.491	0.491	72.227	**<0.001**	**<0.001**
season	1	0.002	0.002	0.280	0.599	0.628
log wing area × season	1	0.057	0.057	8.393	**0.006**	**0.012**
residuals	52	0.353	0.007			
	**anal vein area (log mm^2^), female**					
log wing area	1	0.569	0.569	66.390	**<0.001**	**<0.001**
season	1	0.064	0.064	7.435	**0.009**	**0.016**
log wing area × season	1	0.039	0.039	4.530	**0.038**	**0.049**
residuals	52	0.446	0.009			
**(c)**	**subcostal vein area (log mm^2^), male**					
log wing area	1	0.325	0.326	94.876	**<0.001**	**<0.001**
season	1	<0.001	<0.001	0.005	0.941	0.941
log wing area × season	1	0.015	0.015	4.523	**0.039**	**0.044**
residuals	45	0.154	0.003			
	**cubital vein area (log mm^2^), male**					
log wing area	1	0.198	0.198	36.844	**<0.001**	**<0.001**
season	1	0.034	0.034	6.224	**0.016**	**0.021**
log wing area × season	1	0.081	0.081	15.057	**<0.001**	**<0.001**
residuals	45	0.242	0.005			
	**anal vein area (log mm^2^), male**					
log wing area	1	0.323	0.323	41.502	**0.001**	**<0.001**
season	1	0.157	0.157	20.145	**<0.001**	**<0.001**
log wing area × season	1	0.051	0.051	6.497	**0.014**	**0.021**
residuals	45	0.350	0.008			

### 3.2. Wing vein allometries are seasonally plastic, and cubital and anal veins in dry season males do not scale with the wing size

Unlike VO, the three wing veins were sexually and seasonally dimorphic in the absolute size and varied in their relative sizes. Subcostal, cubital and anal veins are larger in females than in males (*t*‐test; *p* < 0.001 for all veins) and in DS compared with WS butterflies (*t*‐test; subcostal: *p* = 0.006; cubital: *p* = 0.001; anal: *p* < 0.001). Anal veins are also relatively larger in females than in males, and in DS than in WS butterflies for individuals with the same wing size (figure 2*d*, table 2a; *p* (FDR) < 0.001 for both comparisons). Subcostal and cubital veins are larger in WS males compared with WS females (figure 2*b*,*c*; electronic supplementary material, table S2; subcostal: *p* (FDR) = 0.026; cubital: *p* (FDR) = 0.042); these same veins are smaller in DS males relative to their DS female counterparts, though not significantly so (figure 2*b*,*c*; electronic supplementary material, table S2). This results in a significant interaction between sex and seasonal form for both veins (figure 2*b*,*c*, table 2a; subcostal: *p* = 0.008, *p* (FDR) = 0.015; cubital: *p* = 0.011, *p* (FDR) = 0.018). DS females have significantly larger subcostal and cubital veins for the same wing size than WS females (figure 2*b*,*c*; electronic supplementary material, table S2; subcostal: *p* (FDR) = 0.032; cubital: *p* (FDR) = 0.005).

Most wing veins in WS and DS butterflies scale with the wing size, but cubital and anal veins in DS males lack this scaling relationship. Larger wings have larger vein areas in general (table 2a; *p* (FDR) < 0.001 for all veins), but all three veins in DS forms scale less steeply with the wing size than in WS forms (figure 2*b–d*, table 2a; subcostal: *p* = 0.040, p (FDR) = 0.051; cubital: *p* < 0.001, p (FDR) < 0.001; anal: *p* = 0.002, *p* (FDR) = 0.005). This effect was particularly pronounced in DS males, where cubital and anal veins stayed about the same size regardless of wing size (figure 2*c*,*d*; electronic supplementary material, table S1c,j; cubital: *p* = 0.683, p (FDR) = 0.683; anal: *p* = 0.179, p (FDR) = 0.201).

Overall, these data showed that VO is constrained in size across all forms and that cubital and anal veins in DS males are also constrained and not scaling with the wing size. This led us to investigate the function of hearing in males via impairment of the VO followed by observations of male mating behaviour. We did this across both seasonal forms and in both single-pair and male–male competition scenarios.

### 3.3. Impairing the Vogel’s organ affected male courtship behaviour differently in wet season and dry season individuals in pairs

We obtained behavioural data from male *B. anynana* of different seasonal forms for 60 male–female pair trials, half with hearing males (15 DS, 15 WS males) and half with deafened males (15 DS,15 WS males). Data for individual courtship durations were pooled across trials for a total of 229 male courtship bouts.

In pair settings, DS and WS *B. anynana* males did not differ in their likelihood to engage in courtship. Most *B. anynana* males engaged in courtship within the 2 h experimental period, courting in 57 out of 60 trials. Deafened and hearing WS males courted in a similar number of trials (13 out of 15 hearing WS males courted (86.7%); 14 out of 15 deafened WS males courted (93.3%); Yates’s chi-squared test; χ^2^ = 0, df = 1, *p* = 1); and all deafened and hearing DS males courted in each trial (all 15 males courted in each treatment; Yates’s chi-squared test; χ^2^ = 0, df = 1, *p* = 1).

**Figure 3.**
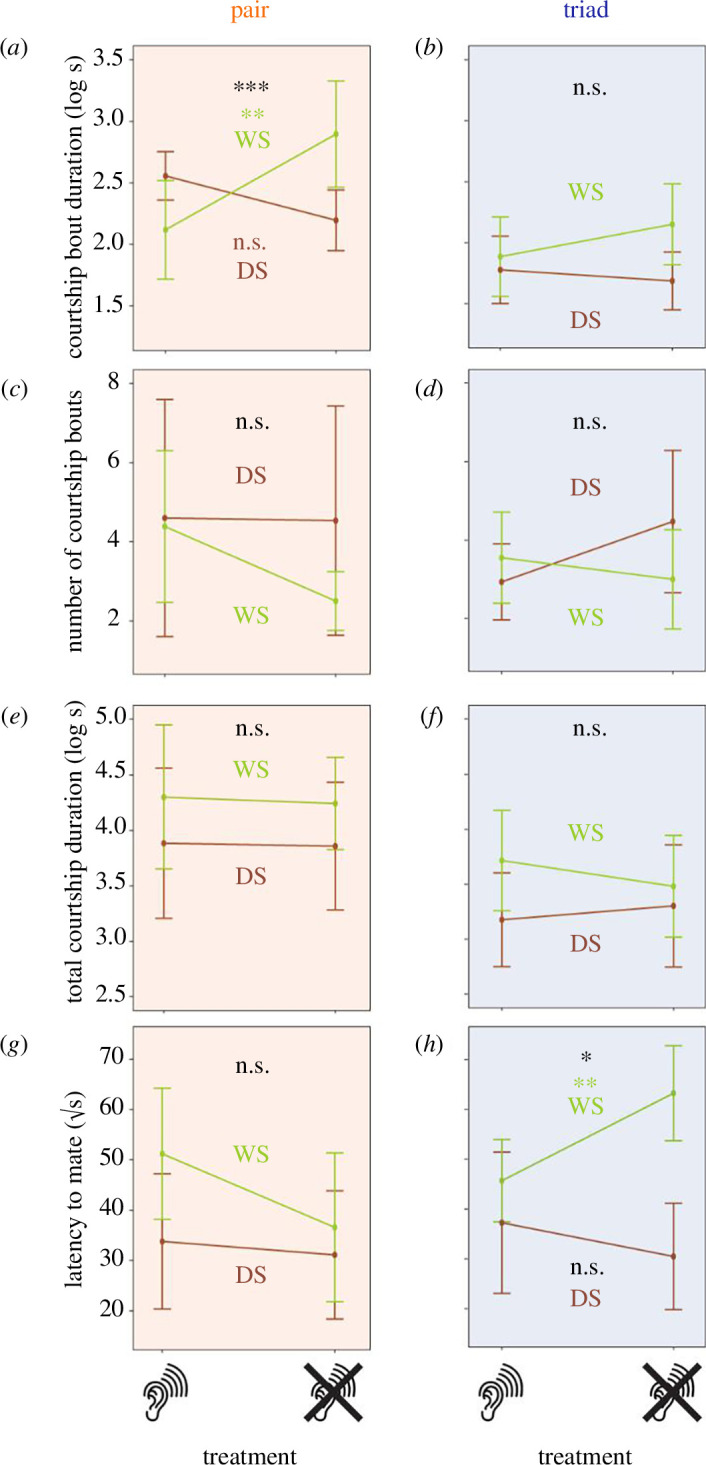
Impairing the VO is associated with seasonally plastic changes in male *B. anynana* courtship behaviour. Graphs depict differences in courtship behavioural metrics between hearing (ear) and deafened (crossed ear) male *B. anynana* in pairs (*a*, *c*, *e*, *g*; *n* = 57) and triads (*b*, *d f*, *h*; *n* = 88), namely (in order) (*a*,*b*) log courtship bout duration, (*c*,*d*) number of courtship bouts, (*e*,*f*) log total courtship duration, and (*g*,*h*) sqrt latency to mate. The number of courtships and total courtship durations plotted here represent only non-zero counts and durations, respectively. Means, reaction norms and 95% confidence intervals are plotted in green for WS and brown for DS. *p*-values for the *treatment* × *season* interaction >0.05, <0.05, <0.01 and <0.001 are represented as ‘n.s.’, ‘*’, ‘**’ and ‘***’, respectively, in black; where the interaction term is significant, *p*-values for the effect of VO impairment on each metric among WS and DS males are represented in green and brown, respectively.

Impairing the VO in males had opposite effects on courtship bout duration in WS and DS males (figure 3*a*, table 2*a*; interaction term: *p* < 0.001); deafened WS males had longer courtship bouts than hearing WS males (figure 3*a*; general linear hypothesis testing, *p* = 0.002), whereas deafened DS males demonstrated marginally (but not significantly) shorter courtship bouts compared with their hearing counterparts (figure 3*a*, table 2*a*; *p* = 0.067, p (FDR) = 0.152).

There were no differences in total courtship duration (courtship duration added across all bouts) and latency to mate between WS and DS seasonal forms, and between deafened and hearing males (figure 3*e*,*g*, table 2*c*,*d*).

### 3.4. In triads, competition changes the effects of Vogel’s organ impairment on male courtship behaviour

Given that male wing flickering may produce sound during courtship, we hypothesized that male*s* use their VOs to listen to the presence of other courting males. As such, we examined how the presence of a male competitor affected courtship behaviour. We obtained behavioural data for 75 triad trials containing 43 DS and 32 WS pairs of hearing and deafened males. Data for individual courtship durations were pooled across trials for a total of 301 courtship bouts.

The presence of a second male had the overall effect of suppressing courtship. Of the males that engaged in courtship, both mean courtship duration (figure 3*a*,*b*) and total courtship duration (figure 3*e*,*f*) in triads were overall lower than in pairs (mean courtship: Wilcoxon rank sum tests; mean courtship duration: *p* < 0.001; total courtship duration: *p* < 0.001). Other measures of courtship, such as mean number of courtships (figure 3*c*,*d*; Wilcoxon rank sum tests: mean number of courtships: *p* = 0.661), mean courtship bout duration, number of courtship bouts and total courtship duration, did not differ across treatments and seasonal forms (figure 3*b*,*f*, table 2e–g).

The presence of a second male, however, had different effects on latency to mate on hearing and deafened males across seasonal forms (figure 3*h*, table 1h). Latency to mate was similar in triads and in pairs for hearing individuals of both seasonal forms (figure 3*g*,*h*), but impairing ears increased latency to mate only in deafened WS males (figure 3*h*; general linear hypothesis testing, *p* = 0.005).

**Figure 4.**
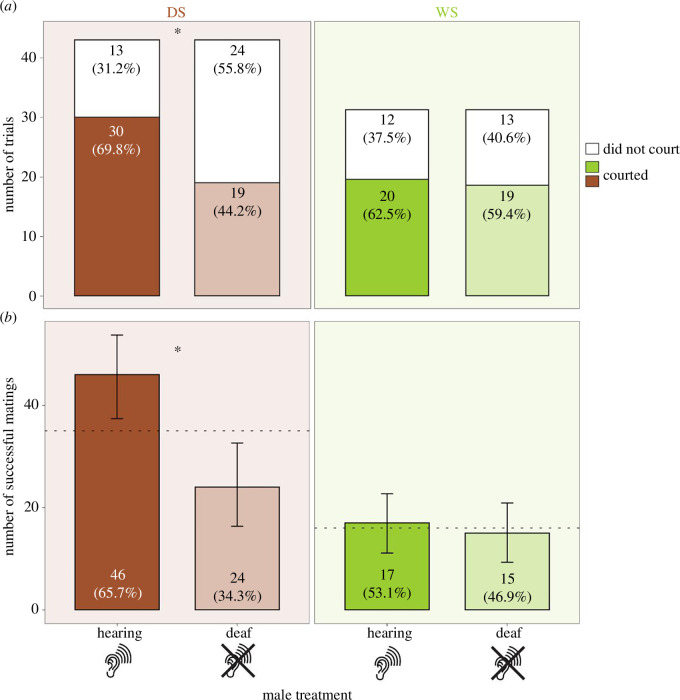
Deafened DS *B. anynana* males are less likely to engage in courtship and less successful at mating compared with hearing males in triads. Graphs depict (*a*) the respective counts and proportions of trials (triads) in which hearing and deafened males engaged or did not engage in courtship among DS (in brown) and WS (in green) males, and (*b*) mating outcomes for DS (in brown) and WS males (in green) in triad settings, with dotted lines at a 50% sample size. *p*-values were derived from chi-squared tests and exact binomial tests for data represented in (*a*) and (*b*), respectively; ‘*’ represents *p*‐value < 0.05. Error bars in (*b*) represent 95% confidence intervals for the exact binomial tests.

Most interestingly, impairing ears affected DS males more than WS males in likelihood of engaging in courtship in triads (figure 4*a*; Yates’s chi-squared test; χ^2^ = 4.744, df = 1, *p* = 0.029), with hearing DS males being more likely to court (30 out of 43 (69.8%) males courted) than deafened DS males (19 out of 43 (44.2%) males courted). There was no difference in courtship likelihood between WS deafened and hearing males (figure 4*b*) (deaf: 19 out of 32 (59.4%) males courted; hearing: 20 out of 32 (62.5%) males courted; Yates’s chi-squared test; χ^2^ = 0, df = 1, *p* = 1).

### 3.5. Vogel’s organ impairment has seasonally plastic effects on mating success

Deafened DS males mated less than hearing males in triad settings, but VO impairment did not impact mating success in WS males. Hearing DS males successfully copulated with the female first in 46 out of 70 trials (figure 4*b*; 65.7%, exact binomial test, *p* = 0.012). On the other hand, hearing WS males achieved first copulation in 17 out of 32 trials (figure 4*b*; 53.1%, exact binomial test, *p* = 0.860).

## 4. Discussion

In this study, we uncovered morphological and allometric features of VO and its flanking wing veins in *B. anynana* that suggest that these organs play an important role in sexual signalling. We also experimentally demonstrated the role of VO in mediating male courtship behaviour, especially in DS forms.

VO and its flanking veins in *Bicyclus anynana* are morphologically constrained, indicating their likely involvement in sound perception. The absolute size invariance of VO and the lack of scaling of the cubital and anal veins of DS males suggest that these traits may be subject to sensory constraints and are evolving under stabilizing selection, as suggested in other insect systems [32,54,55]. VO having a particular size, regardless of wing size, could indicate the optimal size for producing effective resonance in response to specific sound frequencies. Similar auditory constraints are probably shaping the vein areas in *B. anynana*. A previous comparative study showed that the subcostal veins of nymphalid butterflies do not scale with body size in tympanate species but do so in atympanate butterflies [32]. Subcostal veins are also known to increase sensitivity to low-frequency sounds in other satyrine butterflies [25]. Cubital and anal wing veins could be performing a similar function in DS male *B. anynana*. Given that the vein morphological data indicate potentially enhanced hearing sensitivity in DS males and that these males are potentially receptive to female courtship sounds, as they can be courted by DS females [43], we proceeded to investigate the effect of VO impairment in males of both forms.

DS males may use the sounds of competitors as a stimulus to court females. In triad trials, we observed lower mating success in deafened DS males but not in deafened WS males. There was less male courtship across both seasonal forms and increased mating latency in deafened WS males. Although deafened DS males did not change their mating latency, they courted less than their hearing counterparts. This seasonally plastic courtship behaviour translated to reduced mating success in deafened DS males while having no effect in WS males. Our data corroborate previous research that *B. anynana* court and copulate less in the face of competition, in general [56] but also implicate audition in the presence of competition as a driver of courtship behaviour in DS males. The reduced investment in metabolically costly courtship behaviour, given the reduced likelihood of mating success owing to competition, may maximize a male’s net fitness benefits by conserving energy. Perforating the VO, however, suppressed courtship in DS seasonal forms only, suggesting that these males may be listening to the presence of a competitor before investing energy in courting a female. As DS females actively seek out mating opportunities for beneficial male nuptial gifts [43], they may preferentially mate with males that court them sooner. This may explain why hearing DS males had a mating advantage relative to deafened males. The fact that deafened WS males did not alter their courtship behaviour in the presence of a competitor suggests that these males may rely less on sound cues to detect competitors.

Deafened males may invest more in courtship as a response to tissue damage insofar as it increases their chance of mating success. In pair trials, we observed that deafened WS males courted for longer bouts relative to hearing males, whereas this difference was not observed in DS males. Deafened WS males could be courting more in response to perceived damage, increasing their courtship effort as a form of terminal investment for a better chance at mating success. The terminal investment hypothesis proposes that ageing or injured organisms facing reduced survival prospects will increase their allocation of resources into reproduction [57,58]. As courtship is energetically costly [59–61], and longer courtships are usually associated with greater mating success [62–65], longer courtship durations could serve as a useful signal of male fitness to females [62,64,66]. As female *B. anynana* are the choosy sex in the WS [43], WS males can potentially improve their mating success by investing more effort in lengthier courtship displays. This form of terminal investment (in response to perceived damage) makes less sense in DS males, however, because they are often courted by less choosy DS females.

In summary, we show how morphological measurements and manipulative behavioural experiments provide evidence for the role of tympanal ears in sexual communication in diurnal butterflies. We demonstrate that wing tympanal membranes, in addition to functioning in interspecies interactions like predator detection [23,24,26,27], also mediate intraspecies interactions like courtship and male–male competition. Our study is the first, to our knowledge, to experimentally confirm the role of the tympanal ears in altering courtship behaviour in diurnal butterflies. It provides a novel perspective on butterfly communication that has mostly focused on visual and olfactory signals. Discovering a novel mode of communication in charismatic insects, such as butterflies, can impact the way scientists, as well as the public, view and interact with these organisms and inform future policy and conservation strategies. Future research on the nature of the sounds being produced during courtship, the sensitivity of the VO and the enlarged veins to those sounds, the internal morphology and physiology of the wing veins and how they impact the sensitivity of the VO can provide a more complete understanding of auditory communication in butterflies.

## Data Availability

All the data collected during this study and the R scripts are available on the institutional repository of the National University of Singapore, ScholarBank@NUS [67]. Electronic supplementary material is available online [68].
